# Phylogenetic and Mutation Analysis of the Venezuelan Equine Encephalitis Virus Sequence Isolated in Costa Rica from a Mare with Encephalitis

**DOI:** 10.3390/vetsci9060258

**Published:** 2022-05-28

**Authors:** Bernal León, Gabriel González, Alessandro Nicoli, Alicia Rojas, Antonella Di Pizio, Lisbeth Ramirez-Carvajal, Carlos Jimenez

**Affiliations:** 1 LSE Laboratory, Veterinary Service National Laboratory, Animal Health National Service, Ministry of Agriculture and Cattle, Heredia 40104, Costa Rica; 2 Virology, Universidad Técnica Nacional (UTN), Atenas 20505, Costa Rica; 3 National Virus Reference Laboratory, College Dublin, D04 V1W8 Belfield, Ireland; gabo.gonzalez@ucd.ie; 4 Leibniz Institute for Food Systems Biology at the Technical University of Munich, 85354 Freising, Germany; a.nicoli.leibniz-lsb@tum.de (A.N.); a.dipizio.leibniz-lsb@tum.de (A.D.P.); 5 Laboratory of Helminthology, Centro de Investigación en Enfermedades Tropicales, University of Costa Rica, San José 11501, Costa Rica; anaalicia.rojas@ucr.ac.cr; 6 Veterinary Medicine Infection and Immunity, Virology, University of Utrecht, 3584 CS Utrecht, The Netherlands; lizramirez@gmail.com; 7 Laboratory of Virology, Tropical Diseases Research Program (PIET), School of Veterinary Medicine, Universidad Nacional, Heredia 40101, Costa Rica; carlos.jimenez.sanchez@una.ac.cr

**Keywords:** Bayesian, Costa Rica, E1/E2 dimer, evolution, horse encephalitis, mutations, phylogenetic, VEEV, homology modeling

## Abstract

Venezuelan Equine Encephalitis virus (VEEV) is an arboviral pathogen in tropical America that causes lethal encephalitis in horses and humans. VEEV is classified into six subtypes (I to VI). Subtype I viruses are divided into epizootic (IAB and IC) and endemic strains (ID and IE) that can produce outbreaks or sporadic diseases, respectively. The objective of this study was to reconstruct the phylogeny and the molecular clock of sequences of VEEV subtype I complex and identify mutations within sequences belonging to epizootic or enzootic subtypes focusing on a sequence isolated from a mare in Costa Rica. Bayesian phylogeny of the VEEV subtype I complex tree with 110 VEEV complete genomes was analyzed. Evidence of positive selection was evaluated with Datamonkey server algorithms. The putative effects of mutations on the 3D protein structure in the Costa Rica sequence were evaluated. The phylogenetic analysis showed that Subtype IE-VEEV diverged earlier than other subtypes, Costa Rican VEEV-IE ancestors came from Nicaragua in 1963 and Guatemala in 1907. Among the observed non-synonymous mutations, only 17 amino acids changed lateral chain groups. Fourteen mutations located in the NSP3, E1, and E2 genes are unique in this sequence, highlighting the importance of E1-E2 genes in VEEV evolution.

## 1. Introduction

Venezuelan Equine Encephalitis virus (VEEV) is found in tropical and subtropical countries in America and causes a life-threatening illness in equids and humans [1,2,3]. Cases of VEEV were firstly described in Peru in 1932 and Colombia in 1935 [4], but in 1936 it was isolated for the first time in Venezuela [5]. VEEV is a mosquito-transmitted virus of the genus Alphavirus, Togaviridae family. The genome is a non-segmented, positive-sense RNA of ~11.5 kb which is divided into two major domains, the nonstructural domain from the 5′-termini (about two-thirds of the genome) encoding four non-structural proteins followed by the domain encoding five structural proteins [6]. Non-structural proteins (named NSP1 to NSP4) are translated as one or two polyproteins from the genomic RNA itself, and then cleaved into several intermediates that possess distinct important functions. The structural domain is translated as a polyprotein which is processed to generate the envelope glycoproteins (E1–E2), the capsid protein, and the two small polypeptides E3 and 6K [6]. 

VEEV species are taxonomically separated into six subtypes (I to VI) and nine virus species. Viruses of subtype I complex are relevant as veterinary and human pathogens causing potentially fatal encephalitis in both species [7]. Subtype I is classified into four groups, the subtypes IAB and IC are epidemic groups related to periodic outbreaks [8], while ID and IE are considered avirulent with a limited capability to produce outbreaks, Subtype IC is endemic in Panama, and IE is spread from Mexico to Costa Rica [1,9,10]. Despite the fact that it is considered avirulent, the IE subtype was responsible for two outbreaks in Mexico, Chiapas in 1993, and Oaxaca in 1996, affecting 160 horses [9]. Subtype ID produced two young human male deaths in Panama, with clinical signs reported as headache, fever, myalgia, nausea, and vomiting, progressing to delirium, disorientation, restlessness, and coma [11]. 

Due to serological evidence, subtype IE has been considered endemic in the north of Costa Rica since 1970 [1,12]; however, its epidemiological origin remains unknown. An IgG study in 2013 revealed that VEEV is distributed not only in the North of Costa Rica but in the whole country with a seroprevalence of 36% of 217 horses analyzed [13]. In 2015, the brain sample of a mare positive for IgM to VEEV died with encephalitis symptoms; the sample was determined to be positive using Rt-PCR, and the complete genome obtained was confirmed as VEEV subtype IE [14]. In 2016 unfortunately, a child died from VEEV. She lived near Costa Rica’s Southeastern border with Panama, the subtype could not be determined while in 2020 another brain of an equine positive with IgM to VEEV was positive via Rt PCR, this partial sequence (NSP4) also corresponds to the subtype IE. [14]. Given that the subtype IE is considered non-pathogenic, characterizing this sequence is important to better understand why some strains can cause death in horses and humans.

This study aims to reconstruct the phylogeny and the molecular clock of the VEEV subtype I complex, focusing on the complete sequence isolated in the north of Costa Rica in 2015 and how it fits in the evolutionary history of VEEV as well as analyze mutations present in this sequence in comparison with sequences belonging to epizootic or enzootic subtypes.

## 2. Materials and Methods

### 2.1. Selection of Datasets

A total of 114 VEEV sequences with subtypes information, available in the GenBank/DDBJ/EMBL public databases were retrieved. After the sequences were aligned, a maximum likelihood tree was built with Mega X [15] to corroborate the subtype information supplied in these databases. Two polyprotein coding regions of the genomes were concatenated and codon-aligned using Mega X [15].

The concatenated gene sequences were used to generate a phylogenetic tree with IqTree software [16] considering one partition, and 1000 replicates [17] in the Cipres server [18]. This tree was used to evaluate temporal signs in TempEST V1.5.3 [19]. 

### 2.2. Recombination Analysis

Sequences were analyzed by RDP4, version 4.97 [20]. RDP, GENCONV, Chimaera, MaxChi, BootScan, SiScan, 3Seq were selected as detection algorithms for the recombination analysis. A sequence was considered recombinant if this was significantly supported by at least seven algorithms selected in RDP4 (*p* < 0.01). 

### 2.3. Phylogenetic Analyses 

A path sampling test (PS) method [21] in Beast 2.6.0 [22] using 48 steps and chains of 5 million states was used to select the best clock and population-size models. A posterior set of trees was inferred using Beast 2.6.0 [22] with consideration given for a country’s discrete traits when selecting the symmetric model and the remaining sequences after the selection process with a sampling frequency of 10,000 within the 100 million states in the Cipres server [18].

Tracer v 1.7.1 [23] was used to explore the inferred parameters and confirm effective sample sizes (ESS). Maximum clade credibility tree was inferred with the best posterior distribution using mean node heights after discarding the initial 10% of trees as burn-in in TreeAnnotator [24]. 

### 2.4. Selection Analysis

Sequences of genes were aligned with the ClustalW algorithm (codons) using MEGA X [15]. Selection analysis was performed in DataMonkey (https://www.datamonkey.org/ (accessed on 12 January 2020)) with the following algorithms: BUSTED (Branch-Site Unrestricted Statistical Test for Episodic Diversification) [25], FEL (Fixed Effects Likelihood) [26], and aBSREL (adaptive branch-site Random Effects Likelihood) [27,28]. 

### 2.5. Mutations Analysis

The sequences of subtype IE are divided into three main clusters [9], the Panama group (named here as IE-1), the Pacific group (IE-2), and the Caribbean group (IE-3). Mutations in the MK796243 sequence isolated from Costa Rica were compared against the rest of the subtype’s sequences, and also into the subtype IE. We selected three sequences from lineage IE-2, including a Nicaraguan sequence, two sequences from lineage IE-3, and one sequence from IE-1, considered the most recent common ancestor sequence of the subtype IE. Due to the fact that the sequence MEX 1965 KC344446 IE-3, was isolated from a sentinel animal, Mesocricetus auratus, from a virus that was circulating between 1963 and 1965 in Veracruz, Mexico, (before the IE epizootic in 1996), it was considered as the IE wild type (WT) virus.

### 2.6. 3D Structure Analysis

The amino acid sequences of E1 and E2 were submitted to the I-TASSER software [29] to predict the 3D protein structure of the Costa Rican sequence and the WT sequence KC344446. Only one model for each protein was retrieved (C-scores of 1.92 and1.88 for E1 and E2, respectively). The template used to model both proteins is the structure of an enveloped alphavirus Venezuelan Equine Encephalitis Virus (PDB ID: 3J0C) [30]. The sequence identity between E1 and 3J0C (chain A) is 92%, and between E2 and 3J0C (chain B) is 87%. The two obtained structures were aligned to 3J0C to model the E1/E2 dimer. Using the Structure-alignment tool available in Maestro (Schrödinger Release 2020-3) [31], model E1 was superimposed to 3J0C_A and model E2 to 3J0C_B. 

The dimer was then minimized to a derivative convergence of 0.05 kJ/mol-Å using the Polak–Ribiere Conjugate Gradient (PRCG) minimization algorithm, the OPLS2005 force field, and the GB/SA water solvation model implemented in MacroModel (Schrödinger Release 2020-3) [31]. The dimer interface (defined as all the residues within 4 Å from each monomer) was set to be free to move, and the rest of the structure was minimized by applying a force constant of 200 kJ/molÅ2. The E1/E2 dimer was then used as input for the DUET webserver [32] to evaluate the effect of the mutations on protein stability. 

## 3. Results

**Figure 1:**
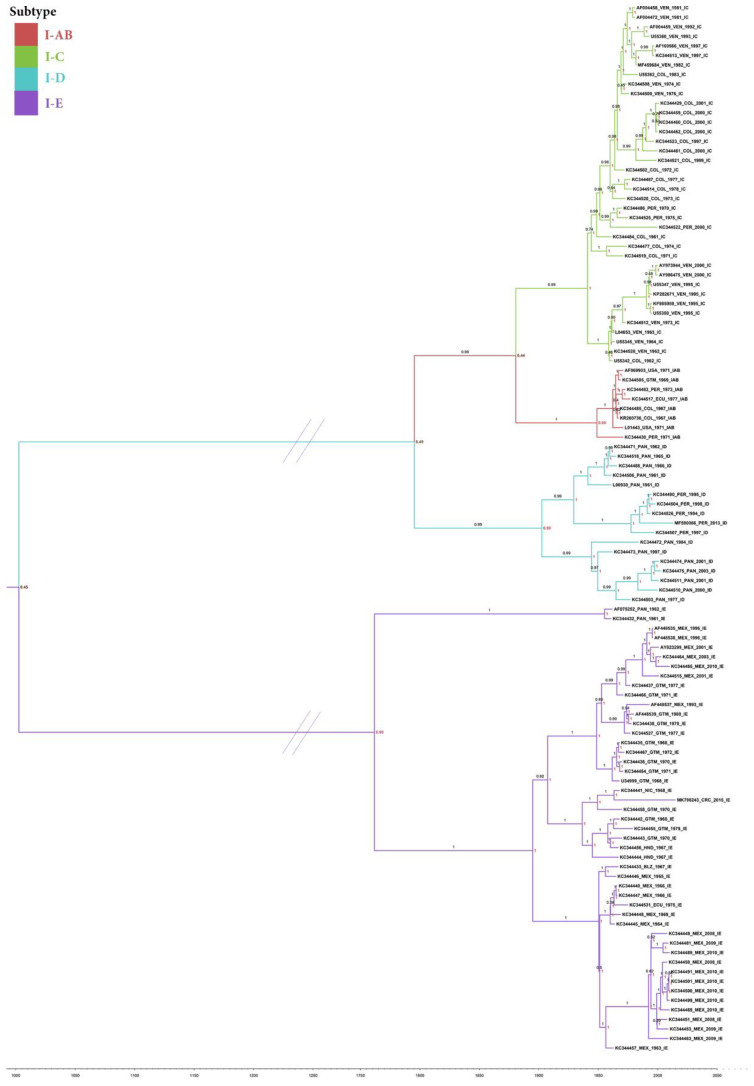
Bayesian inference phylogenetic tree depicting VEEV subtype I topology. Branch labels show the posterior probability, and the node indicates the probability of the subtype origin of the MRCA.

In this study, of the 40 sequences classified in GenBank/DDBJ/EMBL as subtype IC, 22 were considered as ID, and one as IAB subtype (Supplementary Materials and Figure 1), based on the clustering obtained running a tree with Mega X [15]. Of the original 114 sequences, (39 ID, 10 IAB, 17 IC, and 48 in IE), three sequences KC344524_COL_1905_IC, KC344516_VEN_1938_IAB, and L01442_TRI_1943_IAB were discarded because they were outliers which substantially reduced the estimated evolutionary rate before to exclude them (slope = 1.7649 × 10^4^, correlation coefficient = 0.479, R2 = 0.2294, X-Intercept which represents the time to the most recent common ancestor (TMRCA) −400.8497). The sequence KC344493_MEX_IE had recombination in the positions 5428-6222, involving the genes NSP3 and NSP4, with the sequence KC344469_MEX_IE as a major parent, and U34999_GTM_IE as a minor parent (Figure S1, Supplementary Materials), this recombinant sequence was also removed from further analysis. After removing sequences with mismatches between observed and expected dates according to TempEst, and the recombinant sequence, the sampling date was correlated with root-to-tip divergence, slope= 2.125 × 10^4^, correlation coefficient = 0.5036, R2 = 0.2536, X-Intercept (TMRCA) = 6.8594. To corroborate a temporal signal, PS analysis was used to test whether the presence or absence of time information, in a strict clock model, increased the likelihood in the model (20 steps, chains 106 states, burn-in 50%, pre-burn in 105 states). The marginal likelihood estimation (MLE) of the model with no time was −62,114, while the MLE of the model adopting the time data was −61,898, the Bayes factor favored the last one, Table S1. 

Once a temporal signal was confirmed, the most suitable combination of substitution models found by Partitionfinder2 [33], in the remaining 110 concatenate sequences was GTR + I + G4, TVM + I + G4, and GTR + I + G4 for nucleotides in codon positions one, two, and three, respectively, (MLE = −57,840), the relaxed lognormal clock and constant population size were selected with the PS method [21] in Beast 2.6.0 [22] (Table S1, Supplementary Materials).

A phylogenetic tree with the TMRCA among the available sequences was inferred with BEAST. The ESS of all inferred parameters exceeded 200. The posterior probabilities for all branches supporting the topology of the inferred tree are shown in Figure 1. The TMRCA for the considered sequences in subtype I was estimated in the year 1003 (95% HPD; 283–1568) (Figure 1). 

Subtype IE is suggested to have diverged earlier than the rest of the subtypes, the TMRCA according to these sequences dated to the year 1762 (95% HPD: 1555–1897); however, the lack of sequences in the other subtypes and the calibration points limit the robustness of this observation. The TMRCA of the groups IE-2 and IE-3 dated from 1895 (95% HPD: 1710–1997) (Figure 1), while the probability of this MRCA coming from Guatemala is 0.55 (Figure S2, Supplementary Materials).

The Costa Rican sequence MK796243 clustered in the group IE-2 with sequences from Mexico and Central America countries, whose TMRCA was estimated to be 1907 (95% HPD: 1870–1942), and the probability that the ancestor came from Guatemala was 0.70. The TMRCA of the Costa Rican and Nicaraguan sequences was 1963 (95%: HPD 1957–1968), and the probability that this virus came from Nicaragua was 0.74 Figure S2, which largely matches the epidemiological characterizations of these sequences. The TMRCA of the group known as the Caribbean (IE-3) was 1950 (95% HPD: 1941–1959) and the probability that this common ancestor came from Mexico was 0.99 (Figure S2, Supplementary Materials).

The TMRCA of the subtypes IAB-IC-ID was 1795, (95% HPD: 1658–1920). After some divergences, the TMRCA of the subtype ID was calculated around 1902 (95% HPD: 1789–2004) and the probability of the MRCA coming from Panama was 0.85. The TMRCA in the subtype IAB was 1948 (95% HPD1930-1963). In the subtype IC, the TMRCA was 1941 (95% HPD 1926–1954), and there is an 0.83 probability that this ancestor came from Colombia. 

### 3.1. Selection and Mutation Analysis

Next, we assessed the evolutionary forces acting over the selected dataset. Table 1 shows the positive selection for each gene obtained using three different methods, BUSTED, FEL, and ABSREL. According to BUSTED algorithms, only two genes NSP2, and NSP4 showed positive selection among all sequences. Only one branch, sequence KC344438_GTM_1978_IE_2 presented positive diversifying selection (one of 163 branches) in the gene NSP3. FEL analysis of all sequences resulted in three positive selections in amino acid sites, two in the NSP3 and one in E1 codons, while all genes presented purifying selection sites (Table 1 and Table 2). In contrast, FEL analysis showed that the sequence MK796243 from Costa Rica had no presented purifying selection sites, but five genes presented two sites with positive selection (NSP2, NSP3, NSP4, E1, and E2). 

In Table 3, we report 26 mutations observed in the MK796243 sequence when compared with five subtype IE sequences. Changes to 17 amino acids result in changes in chemical properties: 2/2 in the NSP1, 4/5 NSP2, 7/12 in the NSP3 protein (45%), 1/3 in E1, and 3/3 (100%) of the E2. Unique mutations are labeled with a π symbol (Table 3). 

### 3.2. Structural Analysis of Variant Residues of Costa Rican Isolate

**Figure 2:**
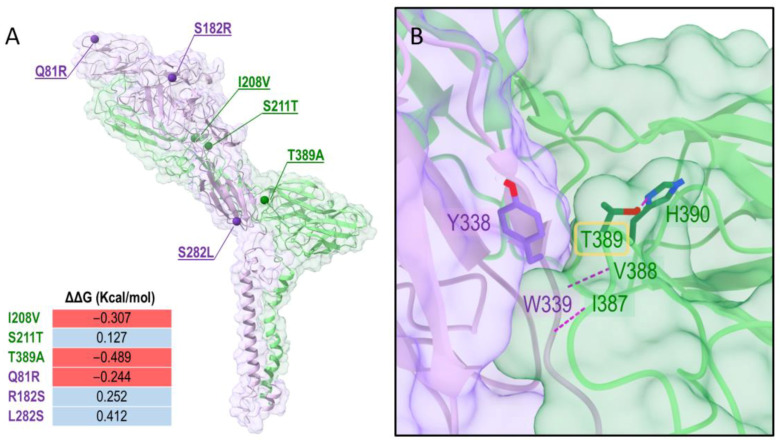
E1/E2 heterodimer model (**A**) and zoomed view of the interface between E1 and E2 at the position T389 (**B**). E1 and E2 are colored in lime green and violet, respectively. Cartoon and transparent surface styles are used. (**A**) Positions affected by mutations in E1 and E2 are shown as dark green and purple spheres, respectively. Calculated ΔΔG values for each mutant are reported. (**B**) Hydrogen bonds are represented as purple dashed lines. Only residues involving side chains in the interactions are shown and labeled: Y338 in E2 and T389 and H390 in E1 are displayed as sticks with atom-type coloring. The images were prepared with ChimeraX [35].

Considering the mutations identified in the previous analysis, we mapped the residue positions that are different in the Costa Rican virus compared to the wild-type sequence subtype MEX 1965 KC344446 IE-3 (Table 3) in the 3D protein context using molecular modeling tools. The E1/E2 3D structure was obtained with a homology modeling procedure. As confirmed also by a BLASTp search [34], the Cryo-EM structure of E1 and E2 of the VEEV TC-83 strain (PDB ID: 3J0C, chains A and B) is currently the only structure available for modeling E1 and E2. E1 and E2 were modeled in their dimeric conformations and the contribution of the mutations to the stability of the dimer was then evaluated with DUET [32]. In Figure 2A, the 3D representation of the dimer and the location of the mutations are shown. The mutations spread through the dimer structure and did not cluster in specific regions. Some mutations were predicted to have a stabilizing effect (i.e., S211T_E1_, R182S_E2_, and L282S_E2_), whereas others had a destabilizing effect (i.e., I208V_E1_, T389T_E1_, Q81R_E2_). Together the mutations may lead to a destabilization of the heterodimer, indeed the overall ΔΔG calculated by summing the individual contributions of E1 and E2 mutations was −0.249 Kcal/mol.

Interestingly, the mutation T389A is predicted to make a major contribution to the destabilization of the dimer. The residue T389 is located in the proximity of the E1/E2 interface, in a central location in an E1 loop interacting with E2: T389 establishes a hydrogen bond with the side chain of H390 of E1 and hydrophobic interactions with Y338 of E2; moreover, it is close to V388 and I387 of E1, which form hydrogen bonds with the backbone of W339 of the E2. This pattern of interactions suggests a key role of T389 in the interaction of the loop where it belongs to E2. A mutation of T389 to alanine might disrupt this network (Supplementary Materials: Figure S3) and increase the flexibility of this loop region, leading to the destabilization of the E1/E2 dimer.

## 4. Discussion

VEEV Subtype I is widely distributed in tropical America, according to our phylogenetic analysis subtype IE has been circulating in Central America since 1764 (596–1771), while subtype IE seems to be the earliest to diverge and the rest subtypes could derive from this. However, this possibility is not well supported (0.45) probably due to the limited number of analyzed sequences, despite the high posterior probability in the phylogenetic tree equal to or higher than 0.95.

Forrester and collaborators (2017) generated two separated trees of the complex I subtype due to the predominance of purifying selection in alphavirus evolution (as was observed in Table 1 and Table 2), which could bias TMRCA estimations for ancient divergence events in RNA viruses [7]. The TMRCA of the ID/IAB/IC strains (Forrester’s first tree) was 1750 (1578–1857), unfortunately, this date could not be accurately determined in their study using the BSREL analysis, and only the TMRCA of the subtype IC could be reliably dated at 1934 (1904–1950) [7], in our case the subtype IC was dated 1941 (1926–1954), while the TMRCA for the ID/IAB/IC group was 1795 (1651–1922), in agreement with their results.

In Costa Rica, the VEEV subtype IE is endemic at least in the north of the country [1,12,14]. Based on serological evidence, Martin et al., (1970) suggested that the VEEV IE viruses were active in the region between 1961–1962 and again in 1967–1968 [1], coinciding with TMRCA of the Costa Rica sequence 1963 (95% HPD 1957–1968).

The ancestor that originated subtype IE could have come from Panama, or a nearby country in South America, and similar to the IAB outbreak in 1969 [1,36], the subtype IE could have spread to North and South America and become endemic in Guatemala, and Mexico, respectively, circulating in reservoirs and vectors in wild cycles. Differences among viral antigens might be correlated with differences in the distribution of their vertebrate hosts or vectors, caused by population migration or evolution.

Subtype IE was previously classified into three distinct lineages named Caribbean, Pacific, and Panama, this classification was based on 868 nucleotides of the precursor E2 (PE2) glycoprotein [9] and the name was given according to the sample geographical distribution. This classification no longer responds to the distribution of some of the sequences based on the complete genome phylogeny obtained in this study, for example, the Costa Rican sequence is located closer to the Pacific coast (named here as IE-2) instead of the Caribbean coast, while the Belize sequence was located in our tree in the IE-3 cluster which would correspond to the Pacific group based on the classification of the E2 glycoprotein, two sequences isolated in Guatemala in 1968 were classified in the groups Caribbean (KC344442) and Pacific (U3499), however, both were clustered into the group IE-2 (complete genome). Therefore, this nomenclature becomes confusing and ambiguous as new samples are collected and the effects of migration and new introductions make it difficult to keep a proper interpretation of the cluster names; for these reasons, we renamed these lineages for simplicity in groups IE-1, IE-2, and IE-3 (Supplementary Materials, Figure S2).

According to our results, enzootic IE-3 strains have been circulating in Mexico since 1950, while the first outbreak of VEEV in Campeche, Mexico was reported in 1962 [37]. In 1963, the VEEV subtype IE was isolated in a sentinel hamster for the first time in Mexico in Santecomapan, Veracruz [38]; this subtype is considered endemic in Mexico.

Subtype IE was never associated with equine virulence until the 1993 epizootic in Chiapas, Mexico [39]. Since then, these IE groups have caused isolated disease events or even outbreaks from time to time, concomitant with the distribution in the continent of its reservoir the cotton rat *Sigmodon hispidus* or even other rodent species [40]. The evolution of the IE subtype could be associated with a significant evolutionary shift from the rest of the VEEV complex, with an increase in structural protein substitutions that may reflect adaptation to its mosquito vector, suggesting that VEEV is maintained primarily in limited geographic foci with only occasional spread to neighboring countries, probably reflecting the limited mobility of rodent hosts and mosquito vectors [7]. Another possibility is that IE enzootic spillover to equines or humans when drift mutations along the genome, increase the adaptability to mosquitoes that feed on equines or humans causing isolated cases sporadically, or that these mutations could increase the viremia in those hosts producing outbreaks like the reported in Mexico in 1993 and 1996 in Mexico [36]. The sylvatic enzootic cycle is maintained between *Culex (Melanoconion*) spp. mosquito vectors and rodent reservoir hosts, while the epizootic cycle involves several different mosquito species like *Aedes taeniorhynchus* which exploit equines as highly efficient amplification hosts, resulting in equine and human disease [1,41].

Different hypotheses about how enzootic subtypes cause disease as epizootic subtypes have been formulated, all involving mutations that increase virulence [42], by adaptation to mosquitoes feeding on horses or humans [43] or increased replication [42].

FEL analysis showed that the sequence MK796243 from Costa Rica, had no presented purifying selection sites, but five genes (NSP2, NSP3, NSP4, E1, and E2), presented two sites with positive selection. A study using a chimeric virus rA774, a nonvirulent Semliki Forest virus SFV (an African Alphavirus), and the SFV4 variant that causes lethal encephalitis in mice, demonstrated that NSPs are related to alphavirus virulence [44].

Experimental mutagenesis assays on the hypervariable domain (HVD) of the NSP3 suggest that the acquired adaptive mutations had different effects on virus replication and might play a crucial role in virus adaptation to new cellular environments [45], especially in insect vectors [46]. Six of the eleven mutations found in the NSP3 (Table 3) are located in the HVD. Mutations in the HVD region could affect the efficient replication in mosquito cells, and the synthesis of the subgenomic RNA in the carboxy-terminal repeat in the VEEV HVD, which is indispensable for VEEV replication [45].

NSP2 caused an increase in infectious viral titers [47]. In our study, five mutations were found in the NSP2 and two of them D454E and t/s495A (pervasive positive/diversifying selection site according to FEL), while Q75H, the histidine also is present in all the sequences of the IAB, ID, and IC subtypes, and A535T, and H617Y involve a change in the amino acid chemistry. The last three mutations are included in the papain-like cysteine protease, which is responsible for processing the non-structural viral polyprotein [47]. NSP2 alone also induces disruption of host macromolecular synthesis, similar to that of capsid protein [48]. Mutations at the amino terminal of the NSP2 cause an increase in infectious virus titer, suggesting that NSP2 has an undetermined function in virus replication and/or virion formation [47].

On the other hand, E2/E1 heterodimers embedded in the plasma membrane are related to receptor recognition and antibody neutralization, also the capsid protein and a genomic RNA molecule interact with E1/E2 to initiate budding virions. Mutations in these genes have been implicated in increasing virulence [42].

Analysis of the Mexican sequences during the outbreak of 1993 and 1996, implicated positively charged amino acid residues with epizootic VEE emergence [42], Interestingly, the three mutations in the E2 found in this study, Q81R, S182R, both pervasive positive/diversifying selection sites according to FEL, and S282L produce a change in the amino acid properties. In our case two of the three amino acids present in the protein E2 are positively charged. The E2 R81 mutation found in the Costa Rica sequence is shared only with three sequences, two Panamanian sequences of the subtype ID, KC344473 1997, and KC344503 1977, and one of the epizootic subtype IC the Venezuelan KC344508 1974 sequence.

A published study confirmed that a small number of positive-charged amino acid replacements mutations (Arg, Lys) in the envelope genes, five in E2, and a single difference in E1 and E3 can generate higher viremia and encephalitis symptoms, that interestingly, were implicated in the transformation of the enzootic VEEV to the equine amplification-competent phenotype [49]. According to our structural analysis, the mutations in the E1/E2 may lead to a destabilization of the heterodimer which could be implicated not only in the receptor recognition but also in the increment of the phenotypic virulence. Experimental in vitro studies or animal models could give some insights into the effect and specific role of the mutations observed in the Costa Rican virus.

Finally, a 795-recombination fragment was detected in a Mexican sequence subtype IE, between the NSP3- NSP4 regions located in the positions 5428-6222. Evidence of recombination between nucleotides 4800-5830 has been reported previously but it could not be confirmed due to NSP3 high variability, as a result of insertions and deletions observed in this region [7]. Recombinant sequences have also been reported in other Alphavirus, Western equine encephalitis virus (WEEV) is the result of a recombination between Sindbis virus (old world alphavirus which contributes with E3, E2, 6K, E1), and Eastern equine encephalitis virus (EEEV NSP1, NSP2, NSP3, NSP4, C), and could not be an occasional event.

## 5. Conclusions

The TMRCA of subtype I was estimated in the year 1003 (95% HPD; 283–1568). While, the subtype IE dated to the year 1762 (95% HPD: 1555–1897); however, the lack of sequences in the other subtypes and calibration points limits the robustness of these estimations. Mutations present in the Costa Rica sequence in the E1/E2 may lead to a destabilization of the heterodimer leading to phenotypic virulence.

## Figures and Tables

**Table 1:** Positive selection by gene, branch, and site was obtained with three different methods.

	BUSTED	FEL		ABSREL
		PP	PN	
NSP1 535	NF	0	216	NF
MK796243	NF	0	0	NF
NSP2 794	F	0	527	NF
MK796243	NF	2 *	0	NF
NSP3 558 MK796243	NF NF	2 2 *	269 0	F, 1/163 KC344438_GTM_1978_IE_2 NF
NSP4 607	F	0	418	NF
MK796243	NF	2 *	0	NF
CAPSIDE 285	NF	0	157	NF
MK796243	NF	0	0	NF
E1 442	NF	1	234	NF
MK796243	NF	2 *	0	NF
E2 423	NF	0	259	NF
MK796243	NF	2 *	0	NF
E3 59	NF	0	34	NF
MK796243	NF	0	0	NF

NF: positive selection was not found, F: positive selection was found, PP: pervasive positive/diversifying selection, PN: pervasive negative/purifying selection. * the pervasive positive/diversifying selection sites are shown in Table 3.

**Table 2:** Amino acid distribution in sites with positive selection.

		SUBTYPE # Seq					
Site	aa	IAB 8	IC 38	ID 17	IE-1 2	IE-2 25	IE-3 20
**NSP 3/HVD-NSP3**							
343	P				2		
	L					1	12
	S					24	7
	-						1
	N	8	38	16			
	D			1			
	-		1				
389	C					3	
	R				2	22	1
	H						19
	E			1			
	G	8	38	16			
**SP-E1**							
347	A			10	2	25	20
	T	8	37	7			
	S		1				

**Table 3:** Amino acid composition among the sequences.

		Pacific IE-2	Pacific IE-2	Pacific IE-2	Pacific IE-2	Caribbean IE-3	Caribbean IE-3	Panama IE-1
		MK796243 CRC 2015	KC344441 NIC 1968	KC344435 GUA 1968	AF448535 MEX 1996	KC344446 MEX 1965	KC344457 MEX 1963	KC344432 PAN 1961
NSP1 (535aa)								
	120	Sπ	T	A	A	A	A	A
	508	Eπ	A	A	A	A	A	A
NSP2 (794aa)								
	75	H **	Q	Q	Q	Q	Q	Q
	454 *	E	D	D	D	D	D	D
	495 *	Aπ	T	T	S	T	T	T
	535	T	T	A	A	A	A	A
	617	Y	H	H	Y	H	H	H
NSP3 (604aa)								
	65	I **	L	L	L	L	L	I
	344 *	P	P	T	L	T	T	T
	350	Sπ	P	P	P	P	P	P
	356	Sπ	S	P	P	P	P	P
	366	Dπ	E	E	E	E	E	D
	389	Cπ ***	S	S	S	S	S	S
	439	Nπ	T	T	T	T	T	T
	452	K	K	Q	Q	Q	Q	R
	465 *	Gπ	E	E	E	E	E	E
	470	Sπ	T	T	T	T	T	T
	528	Cπ	S	S	S	S	S	S
NSP4 (607aa)								
	105 *	V	L	L	L	L	L	L
	235 *	D	E	E	E	E	E	E
E1 (442aa)								
	208	V **	I	V	V	I	I	I
	211 *	Tπ	S	S	S	S	S	S
	389 *	Aπ	T	T	T	T	T	T
E2 (423a)								
	81 *	Rπ	Q	Q	Q	Q	Q	Q
	182 *	Rπ	S	S	S	S	S	S
	282	Lπ	S	S	S	S	S	S

The colors highlighting amino acids represent changes in chemical properties: yellow, red, purple, and light blue indicate positively charged, negatively charged, hydrophobic, and polar amino acids, respectively, while green is either C, G, or P. The acidic E is changed to the hydrophobic A in position 508 of NSP1, the G is changed to the acidic E in position 479 of NSP3, in position 389 of E1 the hydrophobic A is changed with the polar T and in the 282 in E2 the hydrophobic is changed to the polar S. * the pervasive positive/diversifying selection sites according to FEL. ** Histidine (H) at position 75 of NSP2 is shared among all the sequences of the IAB, ID, and IC subtypes, Isoleucine (I) at position 65 of the NSP3 protein is shared with the 2 sequences IE-1 and all the sequences of the IAB, ID, and IC subtypes, Valine (V) at position 208 of the SPE1 protein is shared with 17 sequences of IE-2 group and all sequences of the IAB, 16/17 ID, and IC subtypes. *** In position 389 of the NSP3, the CR sequence has a C, while the subtypes IAB and IC have a G.

## Data Availability

Upon request from the authors.

## Supplementary Materials

The following supporting information can be downloaded at: https://www.mdpi.com/article/10.3390/vetsci9060258/s1, Figure S1: Recombination observed between the sequences of the subtype IE; Table S1: Marginal likelihood estimation (MLE) and Bayes Factor (BF) were obtained when each variable was analyzed.; Figure S2: Topology of VEEV Subtype I by isolation country. Figure S3: Predicted interactions in the E1 and E2 dimer
