# Supplementary material for: Phylogenetic and Mutation Analysis of the Venezuelan Equine Encephalitis Virus Sequence Isolated in Costa Rica from a Mare with Encephalitis
Source: Vet Sci. 2022 May 28;9(6):258. doi: 10.3390/vetsci9060258 (PMC9229380; doi:10.3390/vetsci9060258)
Supplement: Supplementary file 1 [file vetsci-09-00258-s001.zip › vetsci-1696625-supplementary.pdf]

The following 23 sequences classify in GenBank originally as subtype ID were reconsidered as IC: AF004458, AF004472, AF100566, KC344429, KC344459, KC344460, KC344462, KC344477, KC344486, KC344487, KC344502., KC344508, KC344509, KC344512, KC344514, KC344519, KC344521, KC344522, KC344523, KC344524, KC344525, U55362, and as IAB: KC344517

The remain downloaded sequences were classified as:

Subtype IAB:

AF069903, KC344430, KC344483, KC344485,KC344505, L01443, KR260736

Subtype IC: KC344484, KC344520, KC344528, KF985959, KP282671, L04653, MF459684, U55342, U55345, U55347, U55350, U55360, KC344513, AY973944, AY986475, AF004459, KC344461.

Subtype ID:

L00930, MF590066, KC344471, KC344472, KC344473, KC344474, KC344475,

KC344488, KC344490, KC344503, KC344504, KC344506, KC344507, KC344510, KC344511, KC344518, KC344526.

Subtype IE:

KC344531, KC344527, MK796243, U34999, AF075252, AF448535, AF448537,

AF448538, AF448539, AY823299, KC344432, KC344433, KC344435, KC344436, KC344437, KC344438, KC344440, KC344441, KC344442, KC344443, KC344444, KC344445,KC344446, KC344447, KC344448, KC344449, KC344450, KC344451, KC344453, KC344454, KC344455, KC344456, KC344457, KC344458, KC344462, KC344463, KC344464, KC344465, KC344466, KC344467, KC344469, KC344481, KC344489, KC344491, KC344499, KC344500, KC344501, KC344515.

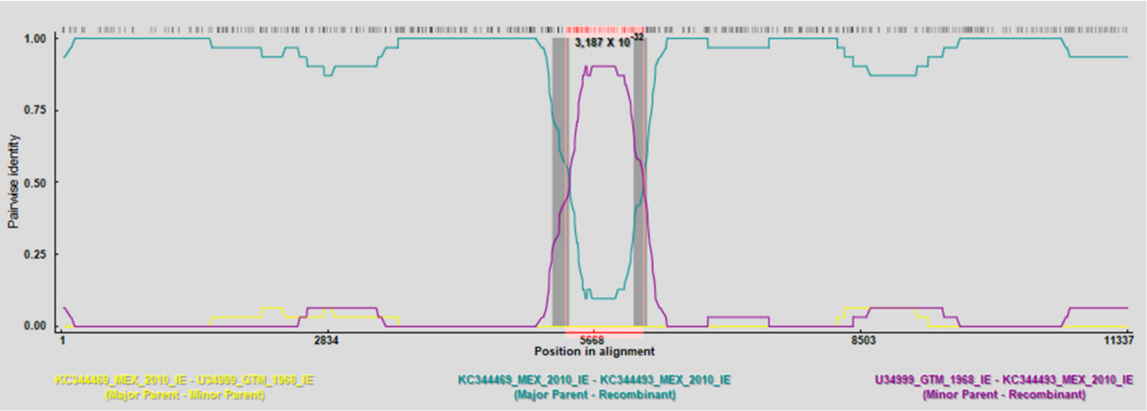

Figure S1. recombination observed between the sequences of subtype IE.

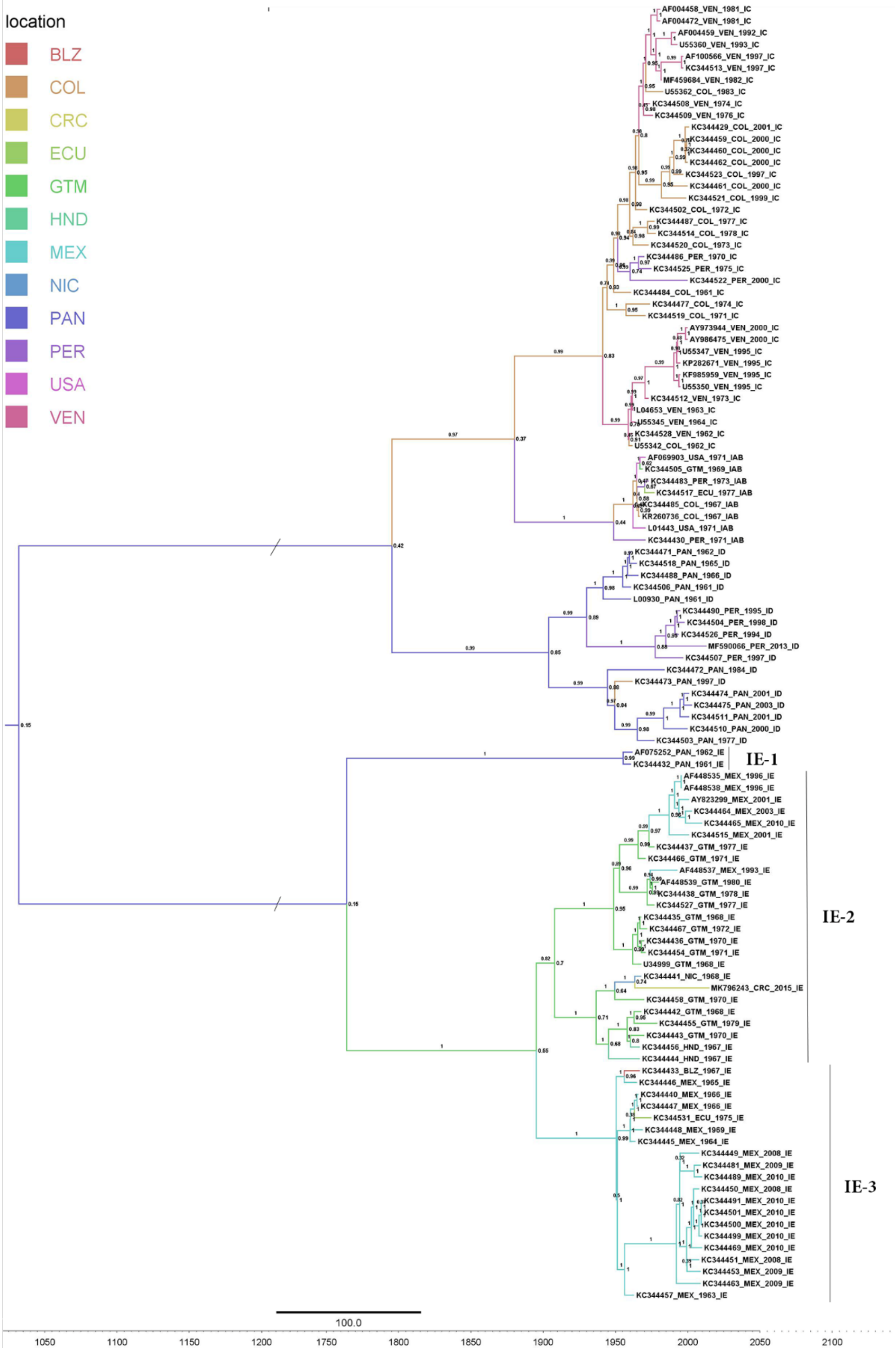

Figure S2. Topology of VEEV Subtype I by isolation country. The node shows the probability of the country of the MRCA, and the branch label depicts the posterior probability, The lines in the Subtype IE show the proposed name for the former lineages Panama, Caribean, and Pacific for IE-1, IE-2, and IE-3.

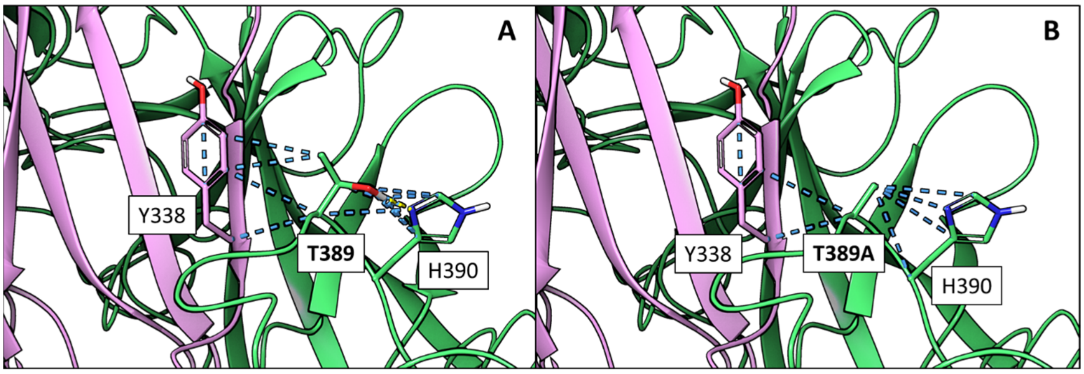

Figure S3. Predicted hydrophobic (dashed blue lines) and H-bond (dashed yellow lines) interactions between T389 (A) and the mutant T389A (B) with surrounding residues. E1 and E2 are depicted as lime green and violet cartoon. Residues sidechain of Y338E2, T389E1, H390E1, and the mutation T389AE1 are displayed as thick tubes with atom-type coloring. The images were prepared with Maestro (Schrödinger).
